# Acute hemoperitoneum after ruptured hepatocellular carcinoma: First Moroccan SCARE-compliant case report and literature review

**DOI:** 10.1016/j.ijscr.2019.12.038

**Published:** 2020-01-11

**Authors:** Rachid Jabi, Badr Sergi, Mehdi Soufi, Soumia El Arabi, Achraf Miry, Tijani El Harroudi, Mohamed Bouziane

**Affiliations:** a Department of General Surgery, Mohammed VI University Hospital, Faculty of Medicine and Pharmacy, Oujda, Morocco; b Department of General Surgery, Ibn Zohr University, Faculty of Medicine and Pharmacy, Agadir, Morocco; c Department of Radiology and Imaging, Mohammed VI University Hospital, Faculty of Medicine and Pharmacy, Oujda, Morocco; d Department of Pathology, Mohammed VI University Hospital, Faculty of Medicine and Pharmacy, Oujda, Morocco

**Keywords:** HCC, Spontaneous rupture, Hepatectomy, Case report, Morocco

## Abstract

**Introduction:**

Acute hemoperitoneum with a spontaneously ruptured hepatocellular carcinoma (HCC) is a rare type of internal bleeding that requires a multidisciplinary management approach.

**Presentation of case:**

In this case report, we describe a fortuitously ruptured peripheral and discovered HCC in an 81-years-old woman treated effectively using hepatectomy.

**Discussion:**

The use of the computed tomography (CT) angiography to diagnose the stability of the patient’s condition allows both arterial embolization in case of active bleeding and the evaluation of the underlying hepatopathy. The place of surgery is particularly upon after both the failure of arterial embolization and while facing a severe hemodynamic instability. The surgical strategy varies between perihepatic tamponnade and a surgical resection in one or more stages. The prognosis of a ruptured HCC depends mainly on the underlying liver’s function.

**Conclusion:**

Multidisciplinary management of spontaneously ruptured HCC results in the same survival outcome as non-ruptured HCC at the same stage of hepatopathy.

## Highlights

• Acute hemoperitoneum after a spontaneously ruptured hepatocellular carcinoma (HCC) is a rare complication.
• In cases with stable conditions, imaging guided by computed tomography (CT) enables arterial embolisation.
• Surgery is a choice after failure of embolisation or in case of severe hemodynamic instability.
• The prognostic outcomes of ruptured HCC depend mostly on the underlying liver function.
• Successful management by one-stage surgery allowed radical treatment of this rare entity.

## 1 Introduction

Ruptured HCC is relatively rare in developed countries and occurs to approximately 3–26% of HCC patients [[1], [2], [3]]. Acute abdominal pain is the major clinical symptom that reveals this association in 97% of cases [4]. It was suggested that tumor necrosis, rapid growth as well as vascular erosion and occlusion of hepatic veins are the most important clinico-pathologic features that influence the intra-tumor pressure and therefore the pathogenesis of this entity [5]. Notably, ruptured HCC leads to hemorrhagic shock which is the main severe issue that influences prognosis [2]. Recent case series consider first-line embolization followed by elective hepatectomy as an effective therapeutic strategy for the management of this fatal entity [6]. However, surgery alone for peripheral lesions of patients with adequate hepatic functions (Child–Pugh score A and B) is also valuable [6]. Moreover, the underlying liver’s function is a key parameter that helps in predicting prognosis of ruptured HCC [7]. We report, to the best of our knowledge, the first case of an acute hemoperitoneum with a ruptured peripheral and fortuitously discovered HCC in an 81-year-old woman managed successfully by a one-stage surgery in Morocco. This work has been reported in line with the SCARE criteria [8].

## 2 Case presentation

**Fig. 1:**
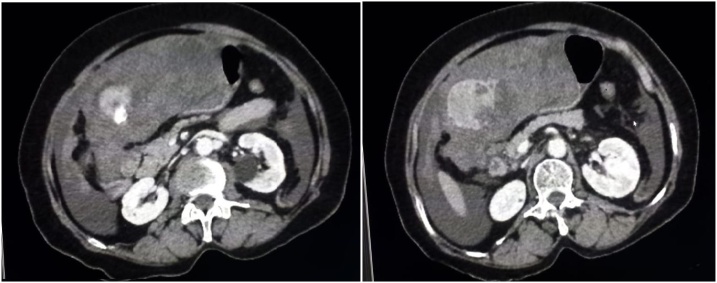
CT scan sections showing a rounded 37.44 mm segment III formation with an heterogeneous enhancement and individualization of an arterial blush with left portal time and hemoperitoneum of high abundance.

**Fig. 2:**
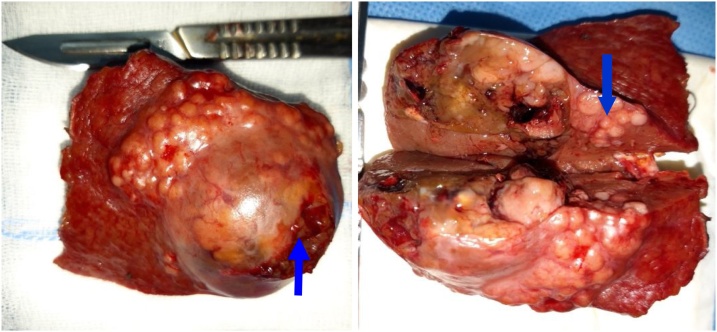
Segmentectomy III surgical specimen (arrows show tumor rupture regeneration nodules).

**Fig. 3:**
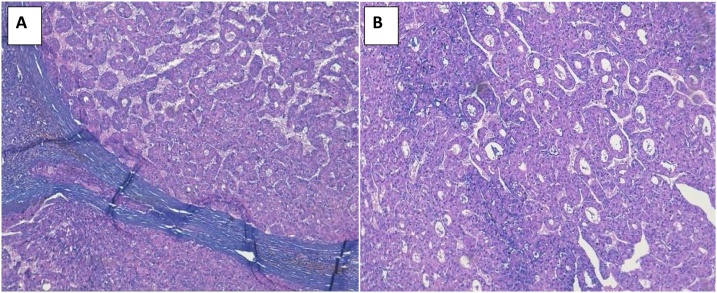
Photomicrographs of the histopathological examination showing carcinomatous proliferations composed by pseudo-glandular formations (A). This proliferation is adjacent to a cirrhotic regeneration nodule (B).

An 81-year-old woman, from eastern Morocco, was admitted to the emergency department following a sudden manifestation of abdominal pain associated with significant distension and increased hypotensive shock with a decrease of blood pressure by 42%. The patient was under oral treatment for diabetes and hypertension and was suffering from no previous diseases. In this regard, laboratory parameters showed hemoglobin level of 5 g/dl and thrombocytopenia of 65,000 elements/μl. Diagnostic ultrasound and CT scan showed a rounded and hyper-vascular nodular formation of 37.44 mm in hepatic segment III with heterogeneous enhancement and individualization of an arterial “blush” which was elevated in the left portal vein that is associated with hemoperitoneum of great abundance (Fig. 1). Following radiological diagnosis, she was transferred to the surgery department for exploration and to maintain the intraoperative hemodynamic stabilization that was ensured at the same time using the transfusion of red blood cells and fresh frozen plasma. Segment III tumor bleeding was detected and 3 liters of blood were collected during laparotomy exploration. Then, a segmentectomy III (Fig. 2) was successful in stabilizing the patient’s hemodynamic status, without undesirable events. Histopathological analysis of the surgical resection specimens revealed a well differentiated HCC (Edmondson–Steiner grade II, pT4) developed on cirrhosis with the presence of a regeneration of liver nodules and unaffected margins (Fig. 3). Postoperative period was smooth and uneventful, and the patient left the hospital on the 5th postoperative day.

## 3 Discussion

According to the latest data from GLOBOCAN 2018, liver cancer, which is dominated by HCC (75%–85% of cases), is the fourth cancer leading to high mortality after lung, colorectal and stomach cancers [9]. In Eastern Morocco (place of origin of our patient), liver cancer is the sixth digestive cancer in terms of incidence [10]. As suggested by a recent systematic review encompassing more than 4900 cases, rupture of HCC is mostly due to patients and tumor specific characteristics including older age, large tumor sizes, portal hypertension and advanced cirrhosis [11]. Acute abdominal pain is the constant sign of ruptured HCC in addition to peritoneal irritation and haemodynamic instability [7]. Acute hemoperitoneum is commonly diagnosed due to ruptured peripherally located HCC such as in our case [7]. Moreover, hypotensive shock may also be seen in a tangential anatomical presentation contrary to central-located HCC in which bleeding is not frequent. In patients with no history of liver diseases, ruptured HCC is often diagnosed during exploratory surgery [4]. Until this time, CT scan still plays a key role in determining the origin of active bleeding and extravasation as well as the location and size of associated hepatic tumors [5,6]. Control of bleeding and shock status by haemostatic procedures and transfusion are required and considered as being major factors to survive after a ruptured HCC. Data from large retrospective cohorts [[11], [12], [13], [14], [15], [16]] (summarized in Table 1) suggest that transarterial embolization (TAE) is the best haemostatic treatment for patients with unstable hemodynamic status. In addition, unstable ruptured HCC patients diagnosed during laparotomy benefit from surgical hepatic resections and artery ligation which are useful homeostatic practices with various rates of in-hospital mortalities [5,[11], [12], [13], [14], [15], [16]]. However, most of these studies reporting single center experiences were retrospective in their design and no randomized and controlled trials were conducted to determine the optimal method for treating ruptured HCC. Our case of ruptured CHC is radiologically suspected in the absence of any known CHC risk factor. After histopathological examination and the confirmation of HCV infection, the patient was treated and recovered favorably. Of note, improved survival of ruptured HCC patients may be achieved after liver tumor resection, and the key factor of poor prognosis is the initial hemodynamic status as well as the underlying liver’s function [6,13]. In conclusion, ruptured HCC should be suspected in patients with acute hemoperitoneum when no risk factor of liver diseases is found.

**Table 1:** Summary of large studies regarding treatment outcomes of ruptured hepatocellular carcinoma.

Author/year	Country	N	Management/objectives	Main findings
Zhong et al. 2016 [12]	China	162	- Analysis of survival outcomes of patients treated with, TAE conservative treatment and partial hepatectomy.	- One month survival is better in patients that undergo hepatectomy or TAE compared with conservative treatment (p < 0.001). - Similarly, long term survival (one year) was achieved in patients treated with either TAE or hepatectomy (p < 0.001). - Notably, higher survival rates were seen in patients treated with partial hepatectomy as compared with those treated with TAE. - On multivariate Cox analysis, TAE and hepatectomy are protective factors for survival (p < 0.001).
Yang et al. 2014 [13]	China	132	- Comparison of five treatment techniques including one- and two-stage surgical resections, conservative treatment, surgical hemostasis, and transarterial embolization (TAE)	- One-stage hepatectomy is a better treatment option for patients with good liver function, while TAE is a better strategy for those with poorly preserved liver function. - No significant difference in terms of median survival was seen between the conservative treatment and surgical hemostasis. - Patients treated with TAE alone had better outcomes as compared with those in the conservative treatment and surgical hemostasis arms. - No significant difference was found between the one-stage and two-stage surgical resection groups.
Aoki et al. 2014 [14]	Japan	1106	- Study of the characteriscs of ruptured HCC and its impact on patients prognosis	- Positive ruptured tumors status is significantly associated with both poor liver functional reserve and advanced tumor status in multivariate analysis. - Analysis of survival outcomes based on TNM staging showed that tumor rupture had an additional impact on tumor staging (addition of 0.5–2 stages to the baseline tumor staging characteristics).
Cheung et al. 2014 [15]	China	189	- Study of prognosis and outcomes of spontaneous ruptured HCC treated by haemostatic RFA (radiofrequency ablation)	- Better overall survival is significantly associated with four independent predictive factors including hemostasis by TAE (HR: 0.516, 95% CI: 0.354–0.751), hemostatic RFA (HR: 0.431, 95% CI: 0.236–0.790), subsequent treatment by surgery (HR: 0.305, 95% CI: 0.186-0.498), and serum total bilirubin level <19 umol/L (HR: 1.596, 95% CI: 1.137–2.241). - Haemostatic treatment using RFA reduces hospital mortality rate as compared with when conventional ligation of hepatic artery.
Zhu et al. 2012 [16]	China	200	- Comparison of survival outcomes of patients treated with hepatic resection, TAE, or conservative treatment	- Selective one-stage hepatectomy improved survival outcomes in patients with resectable tumors.
Zhang et al. 2012 [17]	China	101	- Investigation of short- and long-term survival outcomes in patients treated with hepatectomy.	- Tumor size and blood transfusion quantity were independent risk factors for one moth mortality. - Age, gender, and tumor size were independently significant factors for risk of rupture in multivariate analysis. - Improved survival can be achieved for patients with ruptured tumors after hepatectomy.

N: enrollment.

## Sources of funding

None.

## Ethical approval

Not required for this case report.

## Consent

The patient gave written permission to publish her case findings.

## Author contribution

Dr. Rachid Jabi wrote the manuscript and conducted the literature review. Dr. Soumia El Arabi provided the imaging data of the patient. Dr. Achraf Miry provided the histopathological analysis. Professors Badr Serji, Mehdi Soufi, Tijani El Harroudi, and Mohamed Bouziane supervised the writing of manuscript. All the authors approved the final draft of the paper, for the submission.

## Registration of research studies

Not required.

## Guarantor

Dr. Jabi Rachid (MD).

Pr. Bouziane Mohammed (MD).

## Provenance and peer review

Not commissioned, externally peer-reviewed.

## Declaration of Competing Interest

None.
